# Carotenoids and Liposoluble Vitamins in the Plasma and Tissues of Light Lambs Given Different Maternal Feedings and Fattening Concentrates

**DOI:** 10.3390/ani10101813

**Published:** 2020-10-05

**Authors:** Pablo José Rufino-Moya, Margalida Joy, Sandra Lobón, Juan Ramón Bertolín, Mireia Blanco

**Affiliations:** 1 Centro de Investigación y Tecnología Agroalimentaria de Aragón (CITA), Avda. Montañana 930, 50059 Zaragoza, Spain; pablorufinomoya@gmail.com (P.J.R.-M.); mjoy@aragon.es (M.J.); slobon@cita-aragon.es (S.L.); jrbertolin@cita-aragon.es (J.R.B.); 2 Instituto Agroalimentario de Aragón—IA2 (CITA), Universidad de Zaragoza, 50013 Zaragoza, Spain

**Keywords:** lutein, α-tocopherol, γ-tocopherol, retinol, liver, muscle, fat, authentication, sainfoin, alfalfa

## Abstract

**Simple Summary:**

Meat of lambs that grazed with their dams during lactation on sainfoin had longer shelf-life than that of lambs whose dams grazed on alfalfa or received straw and concentrates (intensive). This effect could be partially ascribed to a different deposition of carotenoids and tocopherols in the muscle, that could be in turn affected by condensed tannins, secondary compounds of plants with antioxidant activity. The objectives of this study were to evaluate: (1) the effect of maternal feeding (sainfoin, alfalfa, intensive) on the presence of carotenoids and liposoluble vitamins in the plasma and tissues of light lambs after a finishing period on concentrates with or without quebracho (as source of condensed tannins); and (2) the authentication of the maternal feeding using the carotenoids and tocopherols in plasma and tissues. Grazing on alfalfa and sainfoin during suckling affected the concentrations in plasma at weaning, and some of the contents in the tissues even after the concentrate feeding period. Unexpectedly, the inclusion of quebracho in the concentrate decreased the α- and γ-tocopherol content in the lamb tissues. The use of carotenoids and tocopherols has to be combined with other analytes to authenticate the feeding system during the suckling period after a finishing period.

**Abstract:**

The carotenoids and liposoluble vitamins in the plasma and tissues of the lambs under different maternal feedings and fattening concentrates was studied. During lactation, 21 lambs were housed with their dams, that received a total mixed ration (intensive); 21 ewe–lamb pairs grazed on alfalfa; and 21 pairs grazed on sainfoin. After weaning, half of the lambs in each maternal feeding group received a commercial concentrate (control) and the other half a concentrate with quebracho (*Schinopsis balansae*), as a source of condensed tannins, until they were slaughtered (23 kg). The analyte concentrations in the plasma of lambs at weaning reflected the content in the feedstuffs. Grazing during suckling more than doubled the contents of lutein in the liver and retinol in the tissues compared to the intensive feeding. The content of α-tocopherol in the tissues was greatest in sainfoin lambs, intermediate in alfalfa lambs, and lowest in the intensive lambs. The quebracho concentrate decreased α-tocopherol (by 41–81%) and γ-tocopherol (by 65–89%) contents in the lamb tissues. The use of the analytes in the plasma at weaning correctly classified 100% of the lambs into the maternal feeding (intensive vs. grazing (alfalfa + sainfoin)) but has to be improved in the carcass and tissues separately.

## 1. Introduction

The traditional lamb meat production systems under Mediterranean conditions have partially or totally replaced grazing using indoor feeding systems, especially during lactation. Lambs are almost exclusively milk-fed until approximately 45 days of age when they are weaned and thereafter, are fed high-concentrate diets until slaughter before 3 months of age. Nowadays, interest regarding grazing systems has increased because such systems are more sustainable than intensive systems [1] and meet consumer demand for products that are considered natural, healthy, and respectful of animal welfare [2]. Nevertheless, the meat quality of grazing lamb is dependent on the forage source as it has been shown that the meat of lambs raised on sainfoin (*Onobrychis viciifolia Scop.*) during suckling has a healthier fatty acid profile, from a human point of view, and a longer shelf life than the meat of lambs raised on alfalfa (*Medicago sativa*) even after a finishing period on concentrates [3]. The meat quality of light lambs has also been modified using quebracho (*Schinopsis balansae*) as a source of condensed tannins (CT); however, the effect is dependent on the diet prior to the fattening period [3,4]. Moreover, the biological effect of CT is dependent on the structure and degree of polymerization. Sainfoin has a medium content of CT, containing procyanidin and prodelphinidin; whereas quebracho contains profisetidin. The structure of the CT of quebracho is more compact and less accessible and consequently the biological effect is lower than that of sainfoin [5,6].

Consumers that purchase products from grass-based systems demand guarantees regarding the diet received by the animals. Carotenoids and tocopherols have been used to authenticate the production system in lambs because these are abundant in fresh forages [7,8], depending the contents on the species, phenological stage or preservation method [9]. These compounds cannot be synthesized by mammals, so they have to be provided in the diet. The content of these compounds in the milk depends on the ewe’s diet [10,11] and they are transferred through the milk to the lamb’s tissues [12,13]. However, the deposition of these compounds in the tissues of lambs depends on several factors such as diet [8], the level of supplementation [14], tissue [15], and interaction between carotenoids and tocopherols [16]. In addition, the antioxidant capacity of CT could protect the carotenoids and liposoluble vitamins from oxidation, thereby allowing a greater deposition in the tissues [17]. The hypothesis of the experiment was that the effect of grazing enhancing the deposition of carotenoids and liposoluble vitamins in the tissues of the suckling lambs might be partially diluted by the post-weaning fattening period. The dilution effect could be milder when quebracho is included in the concentrate due to a possible protection of CT.

The objectives of this study were: (1) to determine the effect of maternal feeding on the presence of carotenoids and liposoluble vitamins in the plasma and tissues of light lambs after a finishing period on concentrates with or without quebracho and (2) the authentication of the maternal feeding using the carotenoids and tocopherols in plasma and tissues of lambs.

## 2. Materials and Methods

The experiment was conducted at the Centro de Investigación y Tecnología Agroalimentaria de Aragón Research Centre Facilities in Zaragoza (41°42′ N, 0°47′ W, 216 m a.s.l.), located in the Ebro Valley (Spain). The experiment and slaughter procedures were conducted in accordance with the requirements of the Spanish Policy for Animal Protection RD *53/2013* (BOE, No. 34, 8 February 2013), which meets the European Union Directive 2010/63/EU for the protection of animals used for experimental and other scientific purposes.

### 2.1. Animal Management and Experimental Design

The present study is part of a broader study that has been described in detail in Lobón, et al. [18]. Briefly, 63 single-bearing Rasa Aragonesa ewes and their male lambs were used in the experiment. The pairs were randomly assigned to one of the following three maternal feedings during the lactation period: (1) intensive: 21 suckling lambs were permanently housed indoors with their dams and received a dry total mixed ration (TMR; neutral detergent fiber (NDF): 42.7%, acid detergent fiber (ADF): 20%, and crude protein (CP): 10.9%) on an ad libitum basis; the pairs were stocked in two pens; (2) alfalfa: 21 suckling lambs were rotationally grazed with their dams on alfalfa (NDF: 45.5%, ADF: 35.2%, and CP: 19.0%); the pairs were stocked in two paddocks; and (3) sainfoin: 21 suckling lambs were rotationally grazed with their dams on sainfoin (NDF: 45.2%, ADF: 31.0%, and CP: 16.1%); the pairs were stocked in two paddocks. The ingredients of the TMR used to feed the ewes in the intensive treatment were barley straw (500 g kg^−1^), corn grain (116 g kg^−1^), barley grain (115 g kg^−1^), alfalfa pellet (93 g kg^−1^), rapeseed meal (70 g kg^−1^), soybean meal (33 g kg^−1^), sugar beet molasses (35 g kg^−1^), cottonseed (14 g kg^−1^), calcium carbonate (19 g kg^−1^), mineral-vitamin corrector (3 g kg^−1^), and salt (2 g kg^−1^).

During lactation, the lambs had continuous access to their dams and free access to a commercial concentrate (control) via a creep concentrate feeder to start the adaptation to the concentrate of the subsequent period. The intake of concentrate of the lambs during the suckling period was 1.3, 1.4 and 4.3 kg DM for sainfoin, alfalfa and intensive treatments, respectively. After weaning (15 ± 0.3 kg Body Weight (BW); 42 ± 2 days of age), the lambs were balanced by BW and allocated in pens, with a total of 12 pens (6 or 5 lambs per pen). The lambs from each maternal feeding group were randomly divided into two treatments with different fattening concentrates: the control concentrate and a concentrate containing 50 g kg−^1^ quebracho (SYLVAFEED ByPro Q, Adial Nutrition. Girona, Spain). The ingredients and chemical composition of the concentrates are reported in Table 1. The lambs received the concentrate and straw ad libitum during 28 (±1.3) days until they reached the target slaughter BW (23 ± 0.1 kg). The total concentrate intake during this period was 22.3 and 23.4 kg dry matter (DM) for the control concentrate and the quebracho concentrate, respectively [18]. Water and mineral blocks were always offered ad libitum.

### 2.2. Measurements and Sampling Procedures

Feedstuffs were sampled weekly. Alfalfa and sainfoin samples were collected by clipping with an electrical mower (3 cm above ground level) in five 0.25 m^2^ quadrats randomly located in the paddock. Part of these samples was oven-dried at 60 °C to a constant weight to obtain the dry matter content and the other part of the samples was freeze-dried to determine the secondary compounds. The freeze-dried samples were ground to pass through a 0.2 mm screen (Rotary Mill, ZM200 Retsch, Haan, Germany) and stored at −80 °C until subsequent analyses.

Lambs were bled from the jugular vein into vacuum tubes containing heparin (Vacuette, Kremsmünster, Austria) at 1 week of age, at weaning and at slaughter. Samples were centrifuged at 3000× *g* at 4 °C for 15 min and the plasma was collected and stored at −80 °C. When the lambs reached the target slaughter weight, they were slaughtered in the experimental abattoir of the Research Centre. After slaughter, the carcasses were chilled at 4 °C for 24 h in total darkness. Then, the *longissimus dorsi* muscle from the first to sixth lumbar vertebrae and the liver were excised and sampled. Muscle and liver samples were lyophilized, vacuum packed, and stored at −80 °C. Samples of the perirenal and caudal subcutaneous fat were obtained and immediately vacuum-packed and frozen at −80 °C until subsequent analyses.

### 2.3. Secondary Compound Analyses

All the samples were analyzed in duplicate.

#### 2.3.1. Extraction of Secondary Compounds in the Feedstuffs

To determine the total polyphenols, 200 mg of the samples were subjected to two successive extractions with 5 mL of acetone:water:formic acid 90% (47.5:47.5:50 *v*:*v*:*v*) [19]. Carotenoids and liposoluble vitamins were extracted in the feedstuffs following the methodologies by Fu, et al. [20] with some modifications. Briefly, 50 mg of forage and 200 mg of concentrates were extracted three times with 3 mL of methanol:acetone:petroleum ether (1:1:1, *v*:*v*:*v*, 0.01% (*w*/*v*) of 2,6-di-tert-butyl-4-methylphenol (BHT)) solution. Then, the supernatant (1 mL for the forage samples and all the supernatant for the concentrate samples) was evaporated. The dry residues obtained in the extractions were dissolved in 1 mL of acetonitrile:dichloromethane:methanol (75:10:15, *v*:*v*:*v*), filtered through a 0.22 µm polytetrafluoroethylene (PTFE) filter, and transferred into a 2 mL glass screw-top vial for automatic sampling using 5 µL for ultra-performance liquid chromatography (UPLC).

Carotenoids and liposoluble vitamins were extracted in the plasma and tissues according to the procedures described by Lyan, et al. [21] and Bertolín, Joy, Rufino-Moya, Lobón and Blanco [15], respectively, with some modifications. Briefly, 1 mL of plasma was deproteinized by 1 mL of ethanol and the lipophilic compounds were extracted twice with 5 mL of n-hexane:ethyl acetate (9:1, *v*:*v*, 5 µg mL^−1^ of BHT) solution. Then, the supernatant was evaporated. In the lamb tissues, the samples were saponified overnight with ascorbic acid and 10% potassium hydroxide (KOH) in ethanol:distilled water (50:50, *v*:*v*) under a nitrogen atmosphere and liquid–liquid extraction with an n-hexane:ethyl acetate (9:1, *v*:*v*, 5 µg mL^−1^ of BHT) solution. Then, the supernatant was evaporated again. The dry residues were dissolved and stored as described for the feedstuff samples.

#### 2.3.2. Analytical Procedures

The extracts of the total polyphenols were analyzed using the Folin–Ciocalteu method following the indications reported in Makkar [19] using tannic acid as the standard. Total CT was analyzed using the method described by Grabber, et al. [22], whereby CT was hydrolyzed to anthocyanidins with 6 mL of butanol:acetone:water:HCl 37% (42:50:3:5 *v*:*v*:*v*:*v*), CO_2_ atmosphere, and 120 min of reaction at 85 °C in a water bath. This was then quantified using extracted and purified sainfoin CT and quebracho CT as standards following the method described by Wolfe, et al. [23].

The chromatographic procedures for the determination of carotenoids and tocopherols in feedstuffs were those used by Chauveau-Duriot, et al. [24], whereas for plasma and animal tissues those described by Bertolín, Joy, Rufino-Moya, Lobón and Blanco [15] were used. For all the determinations, an ACQUITY UPLC H-Class liquid chromatograph (Waters, Milford, MA, USA) equipped with a silica-based bonded phase column (Acquity UPLC HSS T3, 1.8 µm × 2.1 mm × 150 mm column, Waters), an absorbance detector (Acquity UPLC Photodiode Array PDA eλ Detector; Waters), and a fluorescence detector (2475 Multi λ Fluorescence Detector, Waters) controlled by the Empower 3 software was used. Carotenoids and retinol were detected by absorbance at 450 nm and 325 nm, respectively, and tocopherols by fluorescence emission at λ_excitation_ = 295 and λ_emission_ = 330 nm. Carotenoids and tocopherols were identified by the comparison of their retention times and spectral analyses and were quantified by external calibration with pure standards as reported by Blanco, et al. [25]. β-carotene (97% purity), lutein (97% purity), retinol (97.5% purity), and tocopherols (99% purity α-tocopherol, 97% purity γ-tocopherol) were purchased from Sigma-Aldrich (St. Louis, MO, USA).

### 2.4. Calculations and Statistical Analyses

Data were analyzed using the SAS statistical software (SAS V.9.3). The normality of the residues of all variables was tested using the Shapiro–Wilk test. The parameters that did not have a normal distribution of their residues were compared with the Kruskal–Wallis non-parametric test of the NPAR1-WAY procedure.

The content of secondary compounds in the feedstuffs were analyzed with the general linear model (GLM procedure) when the compound had a normal distribution of the residues with the feedstuff of the ewe (alfalfa, sainfoin and TMR)/concentrate of the lamb (control and quebracho) as a fixed effect. The concentration of the liposoluble vitamins in the plasma at the first week and weaning that had a normal distribution of their residues were tested with a mixed model with the maternal feeding (alfalfa, sainfoin and intensive), sampling age (1 week and weaning) and its interaction as fixed effects and the lamb as the random effect. The degrees of freedom were adjusted with the Kenward–Rodgers correction to account for an unequal number of samples or missing values. The concentration of the liposoluble vitamins in the plasma at slaughter and the contents in the tissues of the lamb with a normal distribution of their residues (either with raw data or log-transformed data) were tested using the GLM procedure with the maternal feeding, the inclusion of quebracho in the concentrate, and its interaction as the fixed effects. The least-square means were estimated, and differences were tested with Tukey’s correction. For all tests, the level of significance was set at 0.05. Trends were discussed when *p*-values were < 0.10.

Finally, step-wise selection procedures were used to reduce the number of explanatory variables and a canonical discrimination procedure was used to ascertain the key point variables contributing to a discrimination function of lambs based on carotenoid and liposoluble vitamin content when the entire carcass (the contents in the four tissues, as the lamb can be commercialized in half carcasses or in pieces) was considered. Canonical discrimination procedures were used for each tissue and sampling of plasma separately. A set of analyses was performed considering the three maternal feedings and another set considering intensive vs. grazing (alfalfa and sainfoin) lambs. Canonical correlations with a P-value lower than 0.05 were considered significant.

## 3. Results

### 3.1. Secondary Compounds in Feedstuffs of Ewes and Concentrates of Lambs

The sainfoin contained the highest polyphenols and total CT content, followed by the alfalfa, whereas their content in TMR was almost negligible (Table 2). The TMR had negligible or undetectable carotenoid content, whereas the sainfoin and alfalfa had a high content. They had similar contents except for zeaxanthin and all-β-carotene that were higher in the sainfoin than in the alfalfa (*p* < 0.01). The sainfoin presented the highest α-tocopherol content, followed by the alfalfa and TMR, which presented a low content (*p* < 0.01). Conversely, the TMR had a higher γ-tocopherol content than both forages, which also showed significant differences between them. Regarding the contents of the concentrates fed to the lambs, the inclusion of quebracho in the concentrate increased both the total polyphenols and total CT content (*p* < 0.001). The carotenoids were barely detected or undetectable and γ-tocopherol was the most abundant tocopherol.

### 3.2. Concentration of Carotenoids and Liposoluble Vitamins in the Plasma of Lambs

**Figure 1:**
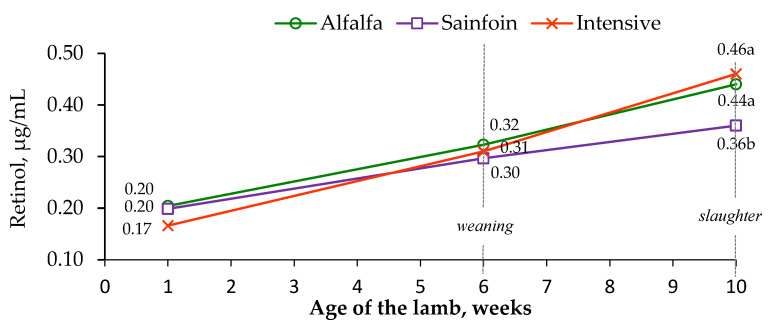
Effect of the maternal feeding on plasma retinol concentrations in lamb. Within an age, means with a different letter differ at *p* < 0.05.

Lutein was only detected at weaning, presenting the lambs on alfalfa and sainfoin similar concentrations (28 and 23 ng/mL, respectively; s.e. = 2.1; *p* = 0.27), whereas lutein was below the detection limit in the lambs on the intensive diet (data not shown). The lambs on the alfalfa and sainfoin diets tended to have greater retinol concentration than the lambs on the intensive diet at the first week of lactation (*p* < 0.10) and attained similar levels at weaning (*p* > 0.05), whereas the lambs on the alfalfa and intensive diets had a higher retinol concentration than the lambs on the sainfoin diet at slaughter (*p* < 0.01; Figure 1).

**Figure 2:**
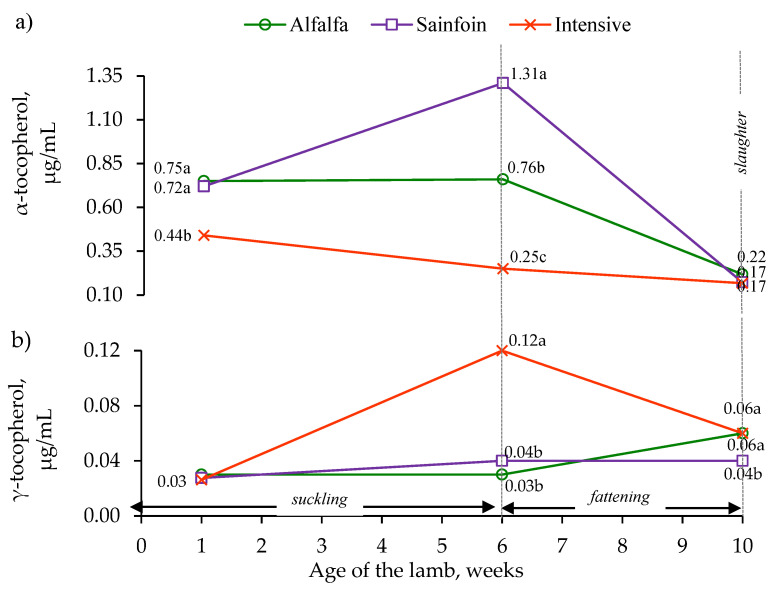
Effect of the maternal feeding on the plasma α-tocopherol (**a**) and γ-tocopherol (**b**) concentrations in the lamb. Within an age, the means with different letter differ at *p* < 0.05.

The α-tocopherol concentration of the lambs on the alfalfa and sainfoin diets was higher than in the lambs on the intensive diet during the first week of lactation (*p* < 0.001). The lambs on the sainfoin diet had the highest concentration of α-tocopherol, the lambs on the alfalfa diet had an intermediate concentration, and the lambs on the intensive diet had the lowest concentration at weaning (*p* < 0.001); however, these differences disappeared at slaughter (Figure 2a). The γ-tocopherol concentration in the first week of lactation was not affected by the maternal feeding (*p* > 0.05); however, it was higher at weaning in the lambs on the intensive diet than those on the alfalfa and sainfoin diets (*p* < 0.05), which had similar concentrations. At slaughter, the lambs on the alfalfa and intensive diets had a higher γ-tocopherol concentration than the lambs on the sainfoin diet (*p* < 0.05; Figure 2b).

The effect of the inclusion of quebracho in the fattening concentrate was only studied on the concentrations at slaughter. At slaughter, the inclusion of quebracho decreased the α-tocopherol concentration (0.21 vs. 0.16 µg/mL, for control and quebracho concentrates, respectively; *p* < 0.05), with no effect on the concentrations of retinol (0.43 vs. 0.41 µg/mL, *p* = 0.93) and γ-tocopherol (0.054 vs. 0.051 µg/mL, *p* = 0.78).

### 3.3. Carotenoid and Liposoluble Vitamin Content in the Lamb Tissues

**Figure 3:**
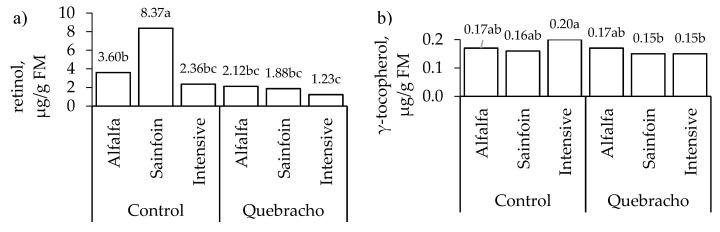
Effect of the maternal feeding and the inclusion of quebracho in the concentrate on the content of retinol in the liver (**a**) and γ-tocopherol in the muscle (**b**). Within a parameter, means with a different letter differ at *p* < 0.05.

The carotenoid and liposoluble vitamin contents were not affected by the interaction between the maternal feeding and the inclusion of quebracho in the concentrate (Table 3) except for the retinol content in the liver and the γ-tocopherol content in the muscle (Figure 3). Therefore, the main results are presented separately except for these two exceptions. In the liver, the lambs on the alfalfa and sainfoin diets had a higher lutein content than that of the lambs on the intensive diet (*p* = 0.001; Table 3). Regarding the retinol content in the liver, the lambs on the sainfoin diet had a higher content than their counterparts when they were fed the control concentrate during fattening (*p* < 0.05); however, there were no differences among the treatments when the lambs were fed the concentrate with quebracho (Figure 3). The maternal feeding did not affect the content of retinol in the muscle (*p* > 0.05) but affected retinol content in fat deposits (*p* < 0.001; Table 3); the lambs on the alfalfa and sainfoin diets had a higher retinol content than the lambs on the TMR diet in both fat deposits (*p* < 0.001). The maternal feeding tended to affect only the α-tocopherol content in the liver (*p* = 0.07) and affected the content in the muscle and both fat deposits (*p* < 0.001). In the three tissues, the lambs on the sainfoin diet had the highest content, the lambs on the alfalfa diet had an intermediate content, and the lambs on the intensive diet had the lowest content (*p* < 0.001).

The inclusion of quebracho reduced the α-tocopherol content in the liver (*p* < 0.001), in the muscle (*p* < 0.05), and in the perirenal fat (*p* < 0.01; Table 3), whereas it decreased the γ-tocopherol content in the liver, in the perirenal fat (*p* < 0.001), and in the subcutaneous fat (*p* < 0.01). In addition, the inclusion of quebracho decreased the retinol content in the liver of the lambs on the sainfoin diet (*p* < 0.001; Figure 3a) and the γ-tocopherol content in the muscle only in the lambs on the intensive diet (*p* < 0.01; Figure 3b).

### 3.4. Relationship between the Concentration of the Analytes in the Plasma and the Content in the Lamb Tissues

The concentration of α-tocopherol in the plasma at weaning was moderately correlated with the content in the muscle (*r* = 0.71; *p* < 0.001) and the perirenal fat (*r* = 0.74; *p* < 0.001) and modestly with the content in the subcutaneous fat (*r* = 0.53; *p* < 0.001). The plasma concentration of the retinol at slaughter was moderately correlated with the content in the muscle (*r* = 0.49; *p* < 0.001).

### 3.5. Discriminant Analysis Based on Carotenoids and Liposoluble Vitamins

The prediction accuracy using the analytes in the plasma at weaning increased from 86% using the samples classified to the maternal feeding (i.e., alfalfa, sainfoin, and intensive) to 100% when alfalfa and sainfoin were grouped as grazing (Table 4). The analytes in the plasma at slaughter correctly classified only 56% of the samples to the maternal feeding and increased to 69% when they were classified as grazing or intensive. Regarding the classification of the entire carcass into maternal feeding, the contents of retinol in the liver and subcutaneous fat, α-tocopherol in the muscle and perirenal fat, and γ-tocopherol in the liver and muscle were selected by the step-wise analyses. These analytes allowed the correct classification of 75% of carcasses to maternal feeding. The percentage of accuracy increased up to 94% when the carcasses were classified into grazing vs. intensive. The use of the analytes in the tissues did not allow for a good classification into their maternal feedings, ranging from 56% to 73%. When the samples from the lambs on the alfalfa and sainfoin diets were grouped into grazing lambs, the percentage of correctly classified samples increased up to 92%.

**Figure 4:**
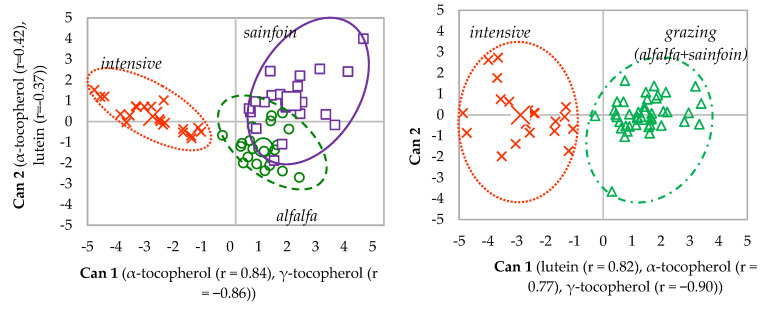
Canonical discriminant plots among maternal feedings in plasma at weaning.

In the discriminant plot of the plasma at weaning, the canonical variables 1 (Can 1) and 2 (Can 2) accounted for 84% and 16% of the total variation among maternal feeding, respectively (Figure 4). When alfalfa and sainfoin samples were grouped, Can 1 accounted for 100% of the total variation among treatments.

**Figure 5:**
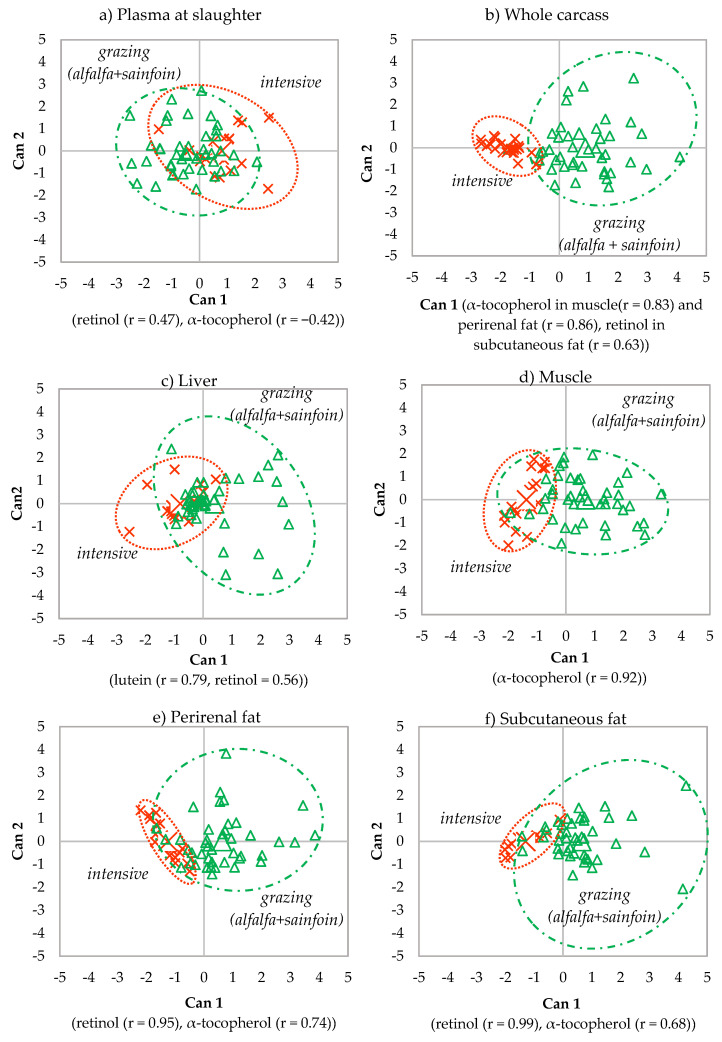
Canonical discriminant plots between maternal feeding (intensive vs. grazing) in the plasma at slaughter (**a**), whole carcass (**b**), liver (**c**), muscle (**d**), perirenal (**e**) and subcutaneous fat (**f**) using the analytes determined in each tissue.

Due to the higher accuracy of the discrimination of the samples of plasma at slaughter, the whole carcass and the tissues when alfalfa and sainfoin lambs were grouped as grazing lambs, only the discriminant plots of grazing vs. intensive feeding during lactation are shown (Figure 5). In all of them, Can 1 accounted for 100% of the total variation between maternal feedings (grazing vs. intensive feeding).

## 4. Discussion

### 4.1. Carotenoids and Tocopherols in the Feedstuffs

The high content of the main carotenes, xanthophylls and α-tocopherol, found in the fresh alfalfa and sainfoin but low or undetectable content found in the TMR and concentrates is in agreement with previous studies that looked at fresh and preserved forages and concentrates [7,8,26]. Concentrates have a low content because of the content in the ingredients and owing to its processing, which involves exposure to high temperatures that lead to the oxidation of carotenoids [9]. In the present study, the high γ-tocopherol content in the TMR could be due to the presence of γ-tocopherol in the rapeseed meal [27] and cotton [28]. The γ-tocopherol content of the concentrates could have been degraded owing to losses during the pelleting procedure (heat, grinding) [29].

### 4.2. Effect of Maternal Feeding on Carotenoids and Liposoluble Vitamins in the Plasma and Tissues of Lambs

In the present study, the concentration of lutein in the plasma at weaning mimicked the content of lutein in the feedstuffs, as has been previously reported in lambs fed on pasture or concentrates [8,26]. Lutein was not detected at slaughter after an indoors fattening period of approximately 28 d, where lambs were fed a concentrate diet, which agrees with the short persistence of carotenoids in plasma of around 14 days [26]. In the present study, the absence of response of retinol concentration of the lambs at weaning to the differences in β-carotene of the feedstuffs and the lower retinol concentration in lambs on the sainfoin diet in comparison with their counterparts at slaughter were unexpected. On the one hand, retinol transport in sainfoin lambs could have been limited, because there is competition among retinol, lutein and tocopherols for the lipoproteins, as reported in cattle with a high intake of carotenoids and tocopherols [10]. On the other hand, retinol concentration in the plasma is highly regulated by deposition in the liver and fat deposits that could have decreased the circulating concentration in the plasma at weaning and during the fattening period [30].

In the current study, the effect of the maternal feeding was quickly reflected in the α-tocopherol concentration in the plasma during the first week of lactation as lambs on the intensive diet had a lower concentration than both groups of grazing lambs owing to the transfer of α-tocopherol in the milk [10,13]. Thus, the plasma α-tocopherol level increased in neonate lambs in response to the supplementation of vitamin E of the ewes during late gestation [31]. At weaning, the tocopherol content in the feedstuffs was reflected in the concentrations of α-tocopherol (sainfoin > alfalfa > intensive) as reported in light lambs by González-Calvo, et al. [32], and γ-tocopherol (intensive > sainfoin > alfalfa). Furthermore, the differences between the lambs on the sainfoin and alfalfa diets might have been enhanced by the protective property of polyphenols over the antioxidant activity of sainfoin from oxidation in the digestive tract. Shabtay, et al. [33] reported that the increase in plasma α-tocopherol in bulls fed with pomegranate peel was not derived from increasing its amount in the diet, but was related to the protective property of polyphenols over antioxidants. From weaning to slaughter, the α-tocopherol concentration in the plasma decreased in lambs on the alfalfa and sainfoin diets with similar concentrations as those of the lambs on the intensive diet at 4 weeks post-weaning, probably due to the low intake of α-tocopherol during the fattening period that led to a depletion of the plasma α-tocopherol concentration. It has been found previously that the α-tocopherol concentration of lambs orally supplemented once with low doses of α-tocopherol acetate (15 and 30 mg/kg live weight (LW)) required 7 days and those supplemented with a higher dose (60 mg/kg LW) required 71 days for the depletion of plasma to attain the same concentration as that of unsupplemented lambs [34]. During the fattening period, the γ-tocopherol concentration in the lambs on the intensive and alfalfa diets changed in response to the content of this analyte in the concentrate. However, the absence of response in the lambs on the sainfoin diet remains to be elucidated.

In the present study, the increased lutein content in the liver in the lambs on the alfalfa and sainfoin diets compared to those on the intensive diet agreed with the content in the feedstuffs. Suckling lambs raised on pasture with their dams were found to have a higher lutein content in the liver than suckling lambs raised indoors with their hay-fed dams [10]. Supporting the previous hypothesis, the higher retinol content in the liver but lower concentration in the plasma of lambs on the sainfoin diet compared to those on the intensive diet was related to a time- and dose-dependent depletion of retinol caused by the concentrate fed during the fattening period before slaughter, as reported in steers [35]. Usually, the α-tocopherol content in the liver reflects differences due to the diet when this diet was fed until slaughter [14,36]. In the present study, the different α-tocopherol content in the maternal feedstuffs was not reflected in the liver of the lambs as has been reported previously in suckling lambs [10], probably due to the fattening period of 28 days when the lambs received only concentrate and straw with low α-tocopherol content. Judson, Babidge and Babidge [34] reported that the α-tocopherol content in the liver of lambs was depleted just 7 days after a single dose of 0, 15, 30, and 60 mg/kg LW α-tocopherol but the content was still high at a higher dose (120 mg/kg LW).

In the muscle, the effect of the maternal feeding on the retinol and γ-tocopherol contents was probably diluted by the concentrate that was fed during the fattening period as shown in the noticeable effect of the maternal feeding on the muscle content observed in suckling lambs [10,12]. Supporting this, the effect of vitamin supplementation on the retinol content in the muscle disappeared after 30 days on a diet with no vitamin A [35]. However, in the present study, the differences in the α-tocopherol content in the muscle owing to the maternal feeding were still evident after the finishing period on concentrates. In addition, the presence of polyphenols or sainfoin CT could also contribute to the α-tocopherol deposition in the muscle. Metabolites of sainfoin CT have been detected in the rumen, abomasum, and small intestine of lambs [37] and, therefore, further research is required to elicit whether they can be deposited in the tissues of lambs. None of the modified signals of the metabolites detected by high-performance liquid chromatography coupled to electrospray ionization and quadrupole time-of-flight mass spectrometry in the polar fraction of the muscle of lambs on alfalfa and sainfoin diets corresponded to the metabolites of proanthocyanidins, but there were differences between them in the metabolites related to the fat metabolic fate [38]. However, further research on the entire metabolome is recommended.

In the present study, the increased retinol content in both fat deposits of lambs on the alfalfa and sainfoin diets compared to lambs on the intensive diet is in agreement with the results observed in the perirenal fat of lambs fed with fresh pasture vs. concentrate [8,39] and in the perirenal and subcutaneous fat of sucking lambs raised indoors with their hay-fed dams vs. those raised on pasture with their dams [10]. The α-tocopherol content in both fat deposits were associated with the content in the feedstuffs as has been reported in previous studies that have shown that the feeding system based in pasture led to higher α-tocopherol content than the concentrate in perirenal and subcutaneous fat of sucking lambs [10] and perirenal fat of light lambs [8]. Similarly, the vitamin E supplementation of diets with different forms has been shown to increase the α-tocopherol content in adipose tissue [14]. As in the muscle, the possible effect of polyphenols or fresh sainfoin CT on the high deposition of α-tocopherol in lambs on a sainfoin diet in the fat deposits compared to lambs on an alfalfa diet remains to be clarified. Regarding the effect of maternal feeding on the γ-tocopherol content in fat deposits, the lambs on the alfalfa, sainfoin, and intensive diets had similar concentrations despite the differences in the γ-tocopherol content in the diets of the ewes. Grazing only has been shown to increase the γ-tocopherol content in the subcutaneous fat of suckling lambs and did not affect the perirenal fat [10] because it is difficult to modify the concentration of γ-tocopherol in the tissues through the diet [30]. Moreover, the finishing period on the concentrates might have meant that the mild differences that could have appeared were not shown.

### 4.3. Effect of the Inclusion of Quebracho on the Contents of Carotenoids and Liposoluble Vitamins in the Plasma and Tissues of Lambs

To the best of our knowledge, there are no previous studies that have evaluated the effect of quebracho on carotenoids and liposoluble vitamins in ovine. The possible effect would be related to the antioxidant effect of polyphenols and CT described in the plasma of ovine [17] and other ruminant species [33]. There are doubts regarding the absorption of CT in the gastrointestinal tract of ruminants. López-Andrés, Luciano, Vasta, Gibson, Biondi, Priolo and Mueller-Harvey [17] suggested that profisetidins of quebracho might not be degraded or absorbed in the gastrointestinal tract of ewes, although there was an improvement shown in the antioxidant capacity of the plasma. In contrast, Gladine, et al. [40] detected epicatechin in the plasma of ewes that were ruminally administered an acute dose of extracts of polyphenols of grape. Then, the effects might be greatly dependent on the source of CT. In the present study, the lower α-tocopherol concentration at slaughter in the plasma of lambs fed the quebracho concentrate because the content of α-tocopherol was greater in aforementioned concentrate and moreover, its intake was also higher than that of the control concentrate [18]. In cows, it has been shown that 1% grape seed and grape marc meal extract, containing CT, increased the retinol concentration in the plasma but did not affect the β-carotene and α-tocopherol content in the plasma of lactating cows [41]. In bulls, a positive effect has been shown for the presence of CT or polyphenols of pomegranate on the α-tocopherol protection in plasma [33]. The aforementioned authors suggested that the polyphenols of pomegranate have protective property over carotenes, α-tocopherol and besides the γ-tocopherol may buffer some degradation of α-tocopherol in the digestive tract, leaving more of it available in the plasma after passage through the liver. In chickens, grape seed supplementation also increased α- and γ-tocopherols in plasma which was explained by the ability of polyphenols to spare/regenerate tocopherol [42]. More studies related to the relationship between the presence of CT and liposoluble vitamins in the plasma of ovine should be undertaken.

The decrease in the retinol and tocopherol contents in the liver with the inclusion of quebracho in the concentrate was unexpected and contradicts previous studies that have shown that phenolic compounds of quebracho induced antioxidant effects in the liver despite not being detected in the organ [17]. Similarly, the decrease in the α-tocopherol content in the muscle of all lambs and only in the γ-tocopherol content in the lambs on the intensive diet with the inclusion of quebracho in the concentrate in the present study contradicted previous studies that used other sources of tannins. The α-tocopherol content in the muscle of lambs increased with rosemary [43], pomegranate subproduct [44], and tara CT [45], or did not change with tannins of red wine extract in lambs [46] and sorghum CT in steers [47]. As in the current experiment, some studies have reported a decrease in γ-tocopherol content with the inclusion of rosemary in lambs [43] and sorghum CT in steers [47]. In contrast, the γ-tocopherol content increased with the inclusion of tara CT [45] and pomegranate [44] due to the higher intake in the diet and presence of polyphenols. Thus, the effect of phenolic compounds on the content of tocopherols depends on the type and dose of compound. More studies related to the influence of CT on the deposition of the carotenoids and tocopherols in the animal tissues should be undertaken.

### 4.4. Discrimination Analysis Based on Carotenoids and Tocopherols

In the present study, the accuracy of the discrimination of the feeding using lutein, retinol, and tocopherols in the plasma at weaning was greater than the accuracy reported by Álvarez, Meléndez-Martínez, Vicario and Alcalde [8] using retinol and α-tocopherol in plasma at slaughter in light lambs. The classification of samples at slaughter obtained in the present study was poor and was unable to discriminate correctly the maternal feeding due to the similar lamb feeding received during the fattening period.

The accuracy of the classification of the animals into their maternal feeding was similar to that of the muscle, perirenal fat, and the entire carcass, and improved when the lambs fed the alfalfa and sainfoin diets were grouped as grazing lambs but did not attain 100% correct classification. However, 100% of the carcasses of suckling lambs were correctly classified into their maternal feeding (hay vs. fresh pasture) using the retinol content of muscle and subcutaneous fat and the γ-tocopherol content of the liver [10]. Carcasses of light lambs after fattening were also well classified according their feeding (grazing vs. concentrate) using retinol, α-tocopherol, and the estimator of carotenoids in fat [8]. Other studies correctly classified 100% of the carcass into their feeding system using the absolute valor of the integral of the translated spectra as estimators of carotenoids of perirenal fat [39,48]. Nevertheless, the discrimination of the lambs in the present study using subcutaneous and perirenal fat color parameters showed lower accuracy than using the content of retinol and tocopherols in both fat deposits because the finishing period on concentrates interfered [18]. Another possibility for tracing the maternal feeding could be the use of fatty acids of the meat, which allowed an 82–100% accuracy in the discrimination among suckling lambs whose dams were fed hay or fresh pasture supplemented with concentrates with or without quebracho [49]. Recently, Prache, et al. [50] concluded that the discrimination can become performance-limited when the methods are used separately and there are often synergies between different methods and tissues, with the authors arguing for combining different tracers (and different tissues for meat products) owing to their observed latency and/or persistent profile differentials. Other secondary compounds such as polyphenol compounds should be considered as biomarkers to increase the reliability of discrimination. Thus, the traceability of the diet in meat under “food quality labels” (i.e., organic production) could be easily implemented using different techniques (NIRS, fat color, carotenoid determination in different fluids and/or tissues) when the lambs receive diets with different carotenoid and tocopherol contents just before slaughter. However, more research is needed before a system combining different techniques can be implemented to guarantee the forage feeding received by the lambs when there is a concentrate feeding period before slaughter, the animals are supplemented concentrates or there are interfering factors such as condensed tannins.

## 5. Conclusions

The carotenoid and tocopherol content from the feedstuffs offered to the ewes were reflected in the contents in the plasma at the weaning of the suckling lambs. The maternal feeding affected the lutein and retinol content in the liver, the α-tocopherol content in the muscle, and the retinol and α-tocopherol content in both fat deposits after a fattening period of 28 days. The observed effect of the inclusion of quebracho on the concentration of tocopherols warrants further research. The use of retinol, lutein, and tocopherols content in the plasma at weaning was an excellent tool to trace the in vivo maternal feeding, since 100% of the animals were accurately classified into grazing or intensive lambs. However, the use of carotenoids and tocopherols should be combined with other metabolites to accurately trace the maternal feeding after a finishing period.

## Figures and Tables

**Table 1:** Ingredients and chemical composition of the concentrates of the lambs.

Item	Control Concentrate	Quebracho Concentrate
Ingredients, g/kg dry matter		
Corn	350	400
Soya bean meal	238	263
Wheat	200	200
Barley	150	20
Quebracho	-	50
Bran	27	-
Calcium carbonate	15	15
Palm oil	12	29
Cane molasses	-	15
Minerals and salt	8	8
Chemical composition, %		
Neutral detergent fiber	17.8	17.7
Acid detergent fiber	4.4	4.1
Crude protein	19.5	21.0

**Table 2:** Contents of carotenoids, tocopherols, total polyphenols and total condensed tannins (CT) in the feedstuffs of the ewes and the concentrates of the lambs.

Item	Ewe’s Feedstuffs					Lamb’s Concentrates			
	TMR ^1^	Alfalfa	Sainfoin	s.e. ^2^	*p*-Value	Control	Quebracho	s.e. ^2^	*p*-Value
Carotenoids									
Neoxanthin, µg/g DM	n.d.	33	28	2.3	0.29	n.d.	n.d.	.	.
Violaxanthin, µg/g DM	n.d.	35	30	.	0.37	n.d.	n.d.	.	.
Zeaxanthin, µg/g DM	n.d.	3.7b	6.1a	.	0.004	0.2	0.3	0.024	0.66
Lutein, µg/g DM	0.2b	111a	127a	7.6	0.001	0.3	0.4	0.024	0.27
13-Z-β-carotene, µg/g DM	0.2b	3.4a	4.1a	.	0.0002	n.d.	n.d.	.	.
9-Z-β-carotene, µg/g DM	n.d.	5.3a	7.2a	0.5	0.06	n.d.	n.d.	.	.
All-β-carotene, µg/g DM	n.d.	31b	65a	4.3	0.0003	n.d.	n.d.	.	.
Tocopherols									
α-tocopherol, µg/g DM	5.4c	35b	89a	4.3	0.001	0.4	1.4	0.159	0.046
γ-tocopherol, µg/g DM	34a	2.9c	5.8b	.	0.001	6.7	4.6	1.0	0.35
Polyphenols, µg tannic acid/g DM	6c	11b	32a	.	0.001	3.8	34	.	0.001
Total CT, eq ^3^/kg DM	n.d.	2.0	25	.	0.001	12	76	2.98	0.001

DM: Dry Matter; n.d.: not detected; ^1^ Total mixed ration given to the ewes indoors in the intensive system; ^2^ not presented for the parameters analyzed with nonparametric models or with transformed data; ^3^ sainfoin equivalents for the ewe’s feedstuffs and quebracho equivalents for concentrates.

**Table 3:** Effect of the maternal feeding during lactation and the concentrate during fattening on the deposition of lutein, retinol and tocopherol in the tissues of the light lamb.

Item	Lactation (L)		Concentrate (C)				*p*-Values		
	Intensive ^1^	Alfalfa	Sainfoin	Control	Quebracho	s.e.	L	C	LxC
n	21	21	21	31	32				
Liver									
Lutein, ng/g FM	1.6b	5.4a	4.9a	4.2	3.1	-	0.001	0.26	0.30
Retinol ^2^, µg/g FM	1.74b	2.79ab	4.19a	4.25a	1.71b	-	<0.001	<0.001	0.01
α-tocopherol, µg/g FM	0.25	0.31	0.30	0.41a	0.17b	-	0.07	<0.001	0.87
γ-tocopherol, µg/g FM	0.14	0.15	0.12	0.17a	0.11b	-	0.36	<0.001	0.27
Muscle									
Retinol, µg/g FM	0.032	0.035	0.033	0.032	0.034	0.001	0.19	0.35	0.96
α-tocopherol, µg/g FM	0.47c	1.00b	1.27a	1.01a	0.82b	0.042	<0.001	0.03	0.06
γ-tocopherol ^2^, µg/g FM	0.17	0.17	0.15	0.18a	0.16b	0.004	0.10	0.006	0.04
Perirenal fat									
Retinol, µg/g FM	0.61b	1.35a	1.43a	1.04	1.16	-	<0.001	0.25	0.17
α-tocopherol, µg/g FM	1.47c	2.51b	3.78a	3.05a	2.11b	0.147	<0.001	0.003	0.30
γ-tocopherol, µg/g FM	1.33	1.23	1.24	1.41a	1.13b	0.041	0.58	0.001	0.69
Subcutaneous fat									
Retinol, µg/g FM	0.48b	1.01a	1.08a	0.80	0.91	-	<0.001	0.27	-
α-tocopherol, µg/g FM	1.37c	2.77b	4.29a	3.15	2.47	0.357	<0.001	0.06	0.34
γ-tocopherol, µg/g FM	1.05	0.98	0.97	1.09a	0.92b	0.030	0.51	0.006	0.88

Within an analyte and effect, the means with a different letter differ at *p* < 0.05; FM: Fresh Matter; ^1^ lambs whose dams were fed a total mixed ration during lactation; ^2^ the interaction is represented in Figure 3.

**Table 4:** Percentages of correctly classified samples of the lambs into the maternal feeding.

Item	Intensive ^1^	Alfalfa	Sainfoin	Intensive ^1^	Grazing (Alfalfa + Sainfoin)
Plasma at weaning	100%	81%	76%	100%	100%
Plasma at slaughter	57%	24%	86%	76%	62%
Carcass	95%	67%	62%	100%	88%
Liver	67%	48%	52%	86%	62%
Muscle	95%	52%	71%	100%	79%
Perirenal fat	67%	62%	86%	86%	86%
Subcutaneous fat	95%	48%	43%	95%	88%

^1^ lamb whose dams were fed a total mixed ration indoors.
